# Reliability of the test of gross motor development: A systematic review

**DOI:** 10.1371/journal.pone.0236070

**Published:** 2020-07-16

**Authors:** Ezequiel Rey, Aida Carballo-Fazanes, Cristina Varela-Casal, Cristian Abelairas-Gómez, on behalf of ALFA-MOV Project collaborators

**Affiliations:** 1 Faculty of Education and Sport Sciences, University of Vigo, Vigo, Spain; 2 Health Research Institute of Santiago de Compostela (IDIS), Santiago de Compostela, Spain; 3 CLINURSID Research Group, Psychiatry, Radiology, Public Health, Nursing and Medicine Department, Universidade de Santiago de Compostela, Santiago de Compostela, Spain; 4 Faculty of Education Sciences, Universidade de Santiago de Compostela, Santiago de Compostela, Spain; Iranian Institute for Health Sciences Research, ISLAMIC REPUBLIC OF IRAN

## Abstract

**Objective:**

To identify, synthesise and evaluate studies that investigated the reliability of the Test of Gross Motor Development (TGMD) variants.

**Methods:**

A systematic search was employed to identify studies that have investigated internal consistency, inter-rater, intra-rater and test-retest reliability of the TGMD variants through Scopus, Pubmed/MEDLINE, PsycINFO, Sport Discus and Web of Science databases.

**Results:**

Of the 265 studies identified, 23 were included. Internal consistency, evaluated in 14 studies, confirming good-to-excellent consistency for the overall score and general motor quotient (GMQ), and acceptable-to-excellent levels in both subscales (locomotor and ball skills). Inter-rater reliability, evaluated in 19 studies, showing good-to-excellent intra-class correlation coefficient (ICC) values in locomotor skills score, ball skills score, overall score, and GMQ. Intra-rater reliability, evaluated in 13 studies, displaying excellent ICC values in overall score and GMQ, and good-to-excellent ICC values in locomotor skills score and ball skills score. Test-retest reliability was evaluated in 15 studies with 100% of the statistics reported above the threshold of acceptable reliability when ICC was not used. Studies with ICC statistic showed good-to-excellent values in ball skills score, overall score, and GMQ; and moderate-to-excellent values in locomotor skills score.

**Conclusions:**

Overall, the results of this systematic review indicate that, regardless of the variant of the test, the TMGD has moderate-to-excellent internal consistency, good-to-excellent inter-rater reliability, good-to-excellent intra-rater reliability, and moderate-to-excellent test-retest reliability. Considering the few high-quality studies in terms of internal consistency, it would be recommend to carry out further studies in this field to improve their quality. Since there is no gold standard for assessing FMS, TGMD variants could be appropriate when opting for a psychometrical robust test. However, standardized training protocols for coding TGMD variants seem to be necessary both for researchers and practitioners in order to ensure acceptable reliability.

## Introduction

Fundamental movement skills (FMS) are considered to be the “building blocks” for more developmentally advanced, complex movements essential for adequate participation in many organised and non-organised games, sports, or other specific physical activity [1–3]. FMS are typical classified into locomotor skills (e.g. running and hopping), manipulative or ball skills (e.g. catching and throwing), and stability skills (e.g. balancing and twisting) [1, 4]. Current evidence suggests that FMS competence is associated with better health outcomes in children and, in addition, this motor proficiency may have a potential role in promoting positive long-term health trajectories across the lifespan [5]. However, mastery of FMS does not emerge naturally [6], and learned exposure and environmental factors seems to play an important role in achieving a proficiency level in the period between early childhood (2–3 years) and later childhood (7–10 years) [7].

In light of previously reported health benefits, instruments used to assess and monitor motor proficiency have gained relevance in physical education over the last decades in order to identify students with motor deficiencies, to describe motor proficiency levels, and to support curricular decisions in schools [8]. FMS assessment tools can be broadly classified into two categories: quantity/product-oriented tests or quality/process-oriented tests [4, 9]. Product-oriented measures quantitatively assess the outcome of the movement (i.e. how far, how high) [10]. On the other hand, process-oriented assessment techniques evaluate the presence or absence of movement patterns demonstrated by a child providing qualitative information on children’s motor competence that can be used for design and planning interventions [9, 11]. Among process-oriented assessment tools, the Test of Gross Motor Development (TGMD) and its variants (Test of Gross Motor Development–Second Edition [TGMD-2] and Test of Gross Motor Development–Third Edition [TGMD-3]) are, probably, the most frequently used technique for measuring FMS proficiency in educational, clinical, and research settings because of their low cost and feasibility [12–15]. The TGMD is a normative and criterion-based assessment designed to qualitatively evaluate the gross motor skill performance of children between the ages of 3 to 10 years and 11 months, with and without disabilities [13–15].

The TGMD is composed of two subscales, locomotor and object control/ball skills, which evaluate six to seven FMS with between three to five performance criteria, depending on skill [14, 15] (Table 1). Child performance is scored with 1 or 0 depending on the presence or absence of such criteria and the final raw scores can be converted into percentile ranks and standard scores. The test results can be used to identify children with gross motor developmental delay [16], to design, plan and evaluate the success of program interventions in FMS development, to assess individual progress, and to serve as an assessment tool in research [14].

**Table 1:** TGMD-2 and TGMD-3 subscales, skills, performance criteria, and scores.

TGMD-2						TGMD-3					
Locomotor			Object Control			Locomotor			Ball Skills		
Skills	Number of performance criteria	Max score	Skills	Number of performance criteria	Max score	Skills	Number of performance criteria	Max score	Skills	Number of performance criteria	Max score
Run	4	8	Two hand strike: stationary	5	10	Run	4	8	Two hand strike: stationary	5	10
Gallop	4	8	Stationary dribble	4	8	Gallop	4	8	Forehand strike: self-bounced	4	8
Hop	5	10	Cath	3	6	Hop	4	8	Stationary dribble	3	6
Leap	3	6	Kick	4	8	Skip	3	6	Cath	3	6
Horizontal jump	4	8	Overhand throw	4	8	Horizontal jump	4	8	Kick	4	8
Slide	4	8	Underhand Roll	4	8	Slide	4	8	Overhand throw	4	8
									Underhand Roll	4	8

*Max*: Maximum.

Reliability can be considered a pre-requisite requirement for clinical, educational and research application of any given measure, even more for field-based measures, such as the TGMD test. In this respect, in recent years, several studies have been published that examined the inter-rater, intra-rater, and test-retest reliability of the TGMD in different population groups, including children with autism spectrum disorder [17], children with attention deficit hyperactivity disorder [18], children with visual impairments [19], children with mental and behavioural disorders [20], and children with intellectual disabilities [21]. Given the increasing amount of scientific evidence on this topic and the extensive application of this assessment tool, a systematic review of the reliability of the TGMD appears to be warranted. Therefore, this study aimed to identify, synthesise and evaluate studies that investigated the reliability of the TGMD and critically appraise and summarise their results. The findings obtained may help clarify the true reliability of this test, and thus provide a valuable resource for practitioners and researchers interested in using the TGMD or interpreting its results.

## Methods

### Search strategy

This comprehensive systematic review was conducted according to Preferred Reporting Items for Systematic Reviews and Meta-Analyses (PRISMA) guidelines [22]. The searches were a combination of MeSH terms and free text words organised into three blocks: terms related to motor development, TGMD and reliability (S1 File). They were conducted through the following databases: Scopus, Pubmed/MEDLINE, PsycINFO, Sport Discus and Web of Science. Our PICO (Population, Intervention, Comparison, Outcomes) question [23] was as follows: Is the TGMD a reliable battery (O) in terms of internal consistency, inter-rater, intra-rater & test-retest reliability (C) to evaluate FMS (I) of pre- & schoolchildren (P)? The search was performed on 08 December, 2019.

### Inclusion and exclusion criteria

The inclusion criteria were stablished in function of our PICO question:

#### Participants

Studies with pre-school (≥3 & <6 years old) and/or schoolchildren (≥6 & <12 years old) participants were included. If data on age diverged between participants, only studies with ≤25% of the sample out of the range 3–11 years old were selected. If only statistics of age were reported, we selected those with mean age inside the range 3–11 and with Mean age + Standard Deviation ≤12. Studies omitting data on age were considered ineligible.

#### Intervention

We included articles in which FMS of pre- and/or schoolchildren were assessed with TGMD or any modified version (TGMD-2 / TGMD-3). Only research studies that embraced all the FMS of the TGMD or all the skills of one of both subscales (locomotor / ball skills) were included.

#### Comparison

We considered investigations that studied internal consistency, inter-rater, intra-rater and test-retest reliability of the TGMD.

#### Outcome

The main outcomes were a) internal consistency and b) inter-rater, intra-rater and test-retest reliability of locomotor, ball skills, overall and gross motor quotient (GMQ). Secondary outcomes were reliability assessment of each skill.

#### Type of study

We included original articles published in English, Spanish or Portuguese (from Portugal & Brazil). No minimum sample size was required.

#### Exclusion criteria

Studies whose principal aim was not to evaluate internal consistency, inter-rater, intra-rater and test-retest reliability of the TGMD were excluded. In this way, those manuscripts with secondary or additional results concerning reliability were not included. Commentary and opinion papers, abstracts, letters to editor, systematic reviews and meta-analysis were also excluded.

### Study selection and data extraction

Both screening and eligibility were independently performed by two authors (E.R & C.A-G) to minimize potential bias. If there were disagreements, a third reviewer (A.C-F) was consulted to reach a decision. Data were independently extracted by two reviewers (A.C-F & C.A-G) based on minimum requirements recommended in the *Inclusion and exclusion criteria* [24], and were then cross-checked.

### Methodological quality

Quality of the studies was evaluated using the COSMIN (COnsensus-based Standards for the selection of health status Measurement INstruments) checklist following the COSMIN guideline for systematic reviews [25, 26], which includes 10 boxes with all standards needed to assess the quality of a study on different specific properties [25]. Boxes 4 and 6 were used in order to assess internal consistency and reliability, respectively. The COSMIN checklist evaluates *design requirements* (1 item for internal consistency & 3 items for reliability), *statistical methods* (1 item for both boxes) and the presence or not of *other important flaws in the design or statistical methods* (in both boxes). According to the COSMIN checklist, each item of each box is rated as *very good*, *adequate*, *doubtful* or *inadequate quality* [26]. The quality of each box corresponds with the lowest rating of any item of the box. The evaluation of risk of bias was appraised by two reviewers (A.C-F & C.A-G) using the tools available in COSMIN website (www.cosmin.nl). If there were disagreements and no consensus after discussion, a third reviewer (E.R) was consulted to reach a decision.

### Manuscripts’ statistics

Due to the large variety of statistical analyses observed in included studies, different reliability statistics classifications have been used. Internal consistency was assessed using Cronbach’s alpha. According to the coefficient alpha size guidelines recommended by George and Malery [27], the following values were used to interpret Cronbach’s alpha: α > 0.9 –Excellent, α > 0.8 –Good, α > 0.7 –Acceptable, α > 0.6 –Questionable, α > 0.5 –Poor, and α < 0.5 –Unacceptable. For inter-rater, intra-rater and test-retest reliability interpretation, a Pearson correlation > 0.80 [28] or ICC > 0.70 or Kappa > 0.70 [25, 28] was rated as “adequate reliability”. Taking into account that ICC was the most used statistic in the included studies, to a more specific classification of reliability, the following ICC classification was used: ICCs less than 0.50, between 0.50 and 0.75, between 0.75 and 0.90, and greater than 0.9 were classified as poor, moderate, good reliability, and excellent reliability, respectively [29]. Finally, for reliability analysis of each skill, ‘adequate reliability’ was operationally defined as ≥ 0.6 for ICCs, defined as the minimum useful level of agreement [30], sufficient for observing human movement for screening purposes [31].

## Results

### Summarize of studies

**Fig 1:**
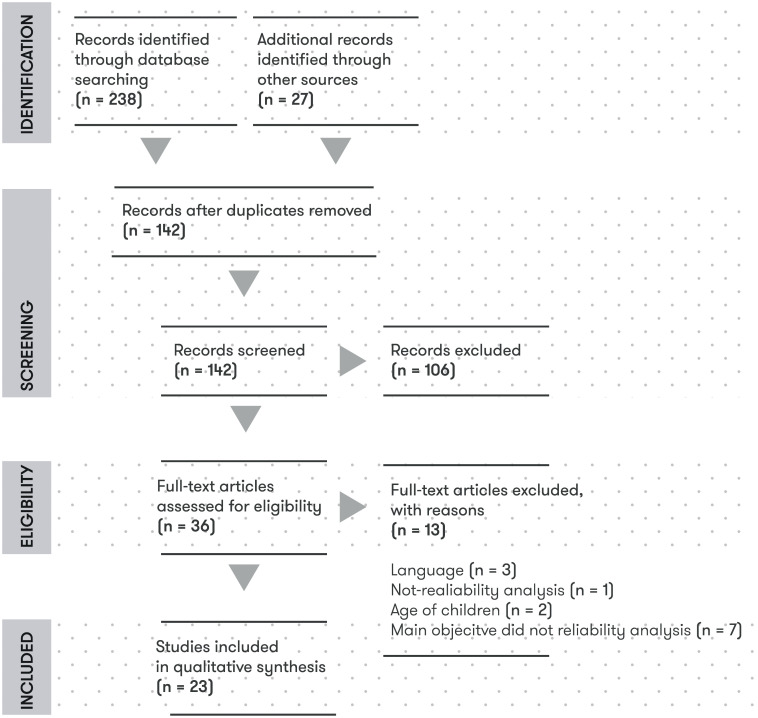
Flow diagram of the search and study selection process.

The initial search retrieved 238 abstracts and 27 additional studies were identified through other resources (i.e. by checking the list of references) (Fig 1). One-hundred and forty-two abstracts were screened after removing duplicates and 23 studies were finally included. There was marked use of TGMD-2 [15 (65.2%)] *vs* TGMD-3 [8 (34.8%)]. Nineteen studies analysed locomotor and ball skills’ score with overall score or GMQ, at least, in one of the reliability measurements (Table 2). Three studies only analysed locomotor and ball skills’ score [32–34] and one only ball skills [35]. In most of cases, video recording was used for evaluating (n = 19). The studies were carried out in 14 different countries with participants aged between 4–9 years and around 40% were girls. Sample sizes ranged from 10 to 2674 participants. Table 3 shows the data extracted from the articles regarding internal consistency, inter-rater, intra-rater and test-retest reliability.

**Table 2:** Summary of the studies included in the review.

First author, year	Test	Country	Sample			Design	
			*N &* Profile	Sex	Age (in years)	Reliability assessment^a^	Viewing type
Allen, 2017 [17]	TGMD-3 traditional protocol	Australia	n = 14 children with ASD	Boys: 10 (71.4)	7.43 (2.03)	Internal consistency	Video evaluation
				Girls: 4 (28.6)		Inter-rater reliability	
	TGMD-3 visual support protocol		n = 21 typically developing children	Boys: 12 (57.1)	7.33 (1.75)	Intra-rater reliability	
				Girls: 9 (42.9)		Test-retest reliability	
Ayán, 2019 [36]	TGMD-2	Spain	n = 84 typically developing children	Boys: 46 (54.8)	8.35 (1.19)	Internal consistency	Video evaluation
				Girls: 38 (45.2)		Inter-rater reliability	
						Test-retest reliability	
Aye, 2017 [37]	TGMD-2	Myanmar	n = 50 typically developing children	Boys: 23 (46.0)	5.40 (0.30)	Inter-rater reliability	Video evaluation
				Girls: 27 (54.0)		Intra-rater reliability (n = 12)	
						Test-retest reliability (n = 25)	
Barnett, 2014 [35]	TGMD-2	Australia	n = 37 typically developing children	Boys: 13 (35.0)	6.20 (0.80)	Inter-rater reliability	Live evaluation
				Girls: 24 (65.0)			
Cano-Cappellacci, 2015 [38]	TGMD-2	Chile	n = 92 typically developing children	Boys: 56 (60.9)	7.50 (1.60)	Inter-rater reliability (n = 32)	Video evaluation
				Girls: 36 (39.1)		Intra-rater reliability (n = 38)	
						Test-retest reliability (n = 32)	
Capio, 2016 [39]	TGMD-2	Philippines	n = 81 children with intellectual disability	Boys: 65 (80.2)	9.29 (2.71)	Internal consistency	Video evaluation
				Girls: 16 (19.8)		Inter-rater reliability (n = 10)	
						Intra-rater reliability (n = 10)	
Estevan, 2017 [40]	TGMD-3	Spain	n = 178 typically developing children	Boys 93 (52.5)	6.94 (1.89)	Internal consistency	Video evaluation
				Girls 85 (47.5)		Inter-rater reliability (n = 4)	
						Intra-rater reliability (n = 4)	
Farrokhi, 2014 [41]	TGMD-2	Iran	n = 1438 typically developing children	Boys: 719 (50.0)	6.53 (2.25)	Internal consistency reliability	Video evaluation
				Girls: 719 (59.0)			
						Intra-rater reliability (n = 32)	
						Test-retest reliability (n = 63)	
Houwen, 2010 [19]	TGMD-2	Netherlands	n = 75 children with visual impairments	Boys: 46 (61.0)	8.50 (1.80)	Internal consistency	Video evaluation
				Girls: 29 (39.0)		Inter-rater reliability (n = 50)	
						Intra-rater reliability (n = 25)	
						Test-retest reliability (n = 23)	
Kim, 2012 [42]	TGMD-2	South Korea	n = 22 children with intellectual disability	Boys: 16 (72.7)	9.90 (1.30)	Inter-rater reliability	Video evaluation
				Girls: 6 (27.3)			
Kim S, 2014 [43]	TGMD-2	South Korea	n = 141 typically developing children	NR	6.80 (1.90)	Internal consistency reliability	Video evaluation
						Inter-rater reliability (n = 40)	
						Test-retest (n = 37)	
Kim C-I, 2014 [44]	TGMD-2	South Korea	n = 121 typically developing children	Boys: 71 (58.7)	5.98 (0.32)	Internal consistency	NR
				Girls: 50 (41.3)			
Lopes, 2018 [45]	TMGD-2	Portugal	n = 330 typically developing children	Boys: 166 (50.3)	7.90 (1.30)	Internal consistency	Video evaluation
				Girls: 164 (49.7)		Inter-rater reliability	
						Test-retest reliability (n = 22)	
Maeng, 2017 [46]	TGMD-3	United States	n = 10 typically developing children	Boys: 6 (60.0)	6.57 (2.51)	Inter-rater reliability	Video evaluation
				Girls: 4 (40.0)		Intra-rater reliability	
Mohammadi, 2019 [47]	TGMD-3	Iran	n = 1600 typically developing children	Boys: 800 (50.0)	6.56 (2.29)	Internal consistency	Video evaluation
				Girls: 800 (50.0)		Inter-rater reliability (n = 160)	
						Intra-rater reliability (n = 160)	
						Test-retest reliability (n = 160)	
Palmer, 2016 [33]	TGMD-2	United States	n = 43 typically developing children	Boys: 25 (57.0)	4.88 (0.28)	Inter-rater reliability	Video evaluation
				Girls: 18 (43.0)			
Rintala, 2017 [48]	TGMD-3	Finland	n = 60 typically developing children	Boys: 28 (46.7)	3–7^b^	Inter-rater reliability (n = 20)	Video evaluation
				Girls: 32 (53.3)		Intra-rater reliability (n = 20 rater A and n = 20 rater B)	
Simons, 2008 [21]	TGMD-2	Belgium	n = 99 children with intellectual disability	Boys: 67 (67.7)	8.83 (1.75)	Internal consistency	Live evaluation
				Girls: 32 (32.3)		Inter-rater reliability	
						Test-retest reliability (n = 8)	
Valentini, 2008 [32]	TGMD-2	Brazil	n = 587 typically developing children	Boys: 300 (51.1)	7.52 (2.04)	Test-retest reliability	Video evaluation
				Girls: 287 (48.9)			
Valentini, 2012 [49]	TGMD-2	Brazil	n = 2674 typically developing children	Boys: 1352 (50.6)	7.56 (1.91)	Inter-rater reliability	Video evaluation
				Girls: 1322 (49.4)		Intra-rater reliability	
						Test-retest reliability (n = 648)	
Valentini, 2017 [50]	TGMD-3	Brazil	n = 597 typically developing children	Boys: 295 (49.4)	Boys: 6.76 (2.11)	Internal consistency reliability	Video evaluation
				Girls: 302 (50.6)	Girls: 6.58 (2.06)	Inter-rater reliability (n = 50)	
						Intra-rater reliability (n = 100)	
						Test-retest reliability (n = 128)	
Wagner, 2017 [34]	TGMD-3	Germany	n = 189 typically developing children	Boys: 99 (52.4)	7.15 (2.02)	Internal consistency	Video evaluation
				Girls: 90 (47.6)		Inter-rater reliability (n = 30)	
						Intra-rater reliability (n = 30)	
						Test-retest reliability (n = 104)	
Webster, 2017 [51]	TGMD-3	United States	n = 807 typically developing children	Boys: 424 (52.5)	6.33 (2.09)	Internal consistency	Live and video evaluation
				Girls: 338 (47.5)		Test-retest reliability (n = 30)	

^*a*^: If sample used in each reliability assessment does not correspond with total sample size, it is specified in brackets.

^*b*^: Age shown as range. Mean and standard deviation of the whole sample size was not reportedsm Spectrum Disorder; *ICC*: Intra-class Correlation Coefficient; *NR*: not reported

*Gender* expressed as absolute frequencies (relative frequencies); *Age* expressed as mean (standard deviation)

**Table 3:** Main results.

First author, year	Test	Reliability results			
		Internal consistency	Inter-rater reliability	Intra-rater reliability	Test-Retest
Allen, 2017 [17]	TGMD-3	ASD Visual support protocol (n = 12):	ASD Visual support protocol (n = 12):	ASD Visual support protocol (n = 12):	ASD Visual support protocol (n = 8):
		LSS: α = 0.93; SEM = 3.28	LSS: ICC = 0.98, 95%CI (0.94–1.00); SEM = 1.75	LSS: ICC = 0.99, 95%CI (0.95–1.00); SEM = 1.24	LSS: ICC = 0.92, 95%CI (0.62–0.98); SEM = 2.99
		BSS: α = 0.81; SEM = 4.39	BSS: ICC = 0.96, 95%CI (0.86–0.99); SEM = 2.02	BSS: ICC = 1.00 95%CI (0.98–1.00); SEM = 0.71	BSS: ICC = 0.83, 95%CI (0.39–0.96); SEM = 2.83
		Overall: α = 0.93; SEM = 5.73	Overall: ICC = 0.99, 95%CI (0.95–1.00); SEM = 2.17	Overall: ICC = 0.99, 95%CI (0.98–1.00); SEM = 2.17	Overall: ICC = 0.92, 95%CI (0.66–0.98); SEM = 4.62
		ASD traditional protocol (n = 14):	ASD traditional protocol (n = 14):	ASD traditional protocol (n = 14):	ASD traditional protocol (n = 8):
		LSS: α = 0.82; SEM = 3.90	LSS: ICC = 0.98, 95%CI (0.92–0.99); SEM = 1.30	LSS: ICC = 0.97, 95%CI (0.88–0.99); SEM = 1.59	LSS: ICC = 0.92, 95%CI (0.65–0.98); SEM = 2.24
		BSS: α = 0.75; SEM = 4.63	BSS: ICC = 0.97, 95%CI (0.91–0.99); SEM = 1.60	BSS: ICC = 0.99, 95%CI (0.96–1.00); SEM = 0.93	BSS: ICC = 0.82, 95%CI (0.31–0.96); SEM = 2.93
		Overall: α = 0.88; SEM = 6.03	Overall: ICC = 0.98, 95%CI (0.94–1.00); SEM = 2.46	Overall: ICC = 0.99, 95%CI (0.92–1.00); SEM = 1.74	Overall: ICC = 0.91 95%CI (0.63–0.98); SEM = 4.06
		Typically developing (n = 21):	Typically developing (n = 21):	Typically developing (n = 21):	Typically developing (n = 17):
		LSS: α = 0.70; SEM = 3.20	LSS: ICC = 0.91, 95%CI (0.79–0.96); SEM = 1.76	LSS: ICC = 0.97, 95%CI (0.93–0.99); SEM = 1.01	LSS: ICC = 0.81, 95%CI (0.54–0.93); SEM = 2.22
		BSS: α = 0.60; SEM = 3.86	BSS: ICC = 0.92, 95%CI (0.81–0.97); SEM = 1.73	BSS: ICC = 0.91, 95%CI (0.68–0.97); SEM = 1.83	BSS: ICC = 0.84, 95%CI (0.62–0.94); SEM = 2.45
		Overall: α = 0.74; SEM = 5.18	Overall: ICC = 0.94, 95%CI (0.87–0.98); SEM = 2.49	Overall: ICC = 0.95, 95%CI (0.84–0.98); SEM = 2.27	Overall: ICC = 0.92, 95%CI (0.78–0.97); SEM = 2.72
Ayán, 2019 [36]	TGMD-2	LSS: α = 0.975	LSS: ICC = 0.976	NR	LSS: r = 0.952
		BSS: α = 0.963	BSS: ICC = 0.956		BSS: r = 0.929
		GMQ: α = 0.974	GMQ: ICC = 0.985		GMQ: r = 0.956
Aye, 2017 [37]	TGMD-2	NR	LSS: ICC = 0.95	LSS: ICC = 0.98	LSS: ICC = 0.82
			BSS: ICC = 0.88	BSS: ICC = 0.95	BSS: ICC = 0.79
			GMQ: ICC = 0.89	GMQ: ICC = 0.97	GMQ: ICC = 0.76
			Rater A x Rater B:		
			LSS: r = 0.97 (p<0.001)		
			BSS: r = 0.96 p<0.001)		
			GMQ: r = 0.97 (p<0.001)		
			Rater A x Rater C:		
			LSS: r = 0.94 (p<0.001)		
			BSS: r = 0.85 (p<0.001)		
			GMQ: r = 0.87 (p<0.001)		
			Rater B x Rater C:		
			LSS: r = 0.93 (p<0.001)		
			BSS: r = 0.96 p<0.001)		
Barnett, 2014 [35]	TGMD-2	NR	BSS: ICC = 0.93 95%CI (0.87–0.96)	NR	NR
Cano-Cappellacci, 2015 [38]	TGMD-2	NR	LSS: CVI = 0.87, 95%CI (0.73–0.93)	LSS: CVI = 0.92, 95%CI (0.83–0.95)	LSS: CVI = 0.86, 95%CI (0.71–0.93)
			BSS: CVI = 0.88 (0.77–0.94)	BSS: CVI = 0.86, 95%CI (0.76–0.93)	BSS: CVI = 0.80, 95%CI (0.59–0.90)
			Overall: CVI = 0.86, 95%CI (0.72–0.93)	Overall: CVI = 0.91, 95%CI (0.83–0.95)	Overall: CVI = 0.88, 95%CI (0.75–0.94)
Capio, 2016 [39]	TGMD-2	LSS: α = 0.830	LSS: ICC = 0.996, 95%CI (0.984–0.999)	LSS: ICC = 0.995, 95%CI (0.978–0.999)	NR
		BSS: α = 0.792	BSS: ICC = 0.998, 95%CI (0.992–1.000)	BSS: ICC = 0.998, 95%CI (0.991–0.999)	
			Overall: ICC = 0.998, 95%CI (0.991–0.999)	Overall: ICC = 0.997, 95%CI (0.989–0.999)	
Estevan, 2017 [40]	TGMD-3	Locomotion: α = 0.80, 95%CI (0.75–0.84)	Overall: ICC = 0.90, 95%CI (0.66–0.98)	Overall: ICC = 0.98, 95%CI (0.85–1.00)	NR
		BSS: α = 0.85, 95%CI (0.81–0.88)			
		Overall: α = 0.89, 95%CI (0.87–0.92)			
Farrokhi, 2014 [41]	TGMD-2	LSS: α* = 0.78	NR	LSS: ICC = 0.95, 95%CI (0.91–0.97)	LSS: ICC = 0.65, 95%CI (0.50–0.79)
		BSS: α* = 0.74		BSS: ICC = 0.99 95%CI (0.97–0.99)	BSS: ICC = 0.85, 95%CI (0.75–0.91)
		GMQ: α* = 0.80		GMQ: ICC = 0.97, 95%CI (0.94–0.98)	GMQ: ICC = 0.81, 95%CI (0.70–0.89)
Houwen, 2010 [19]	TGMD-2	LSS: α = 0.71	LSS: ICC = 0.82, 95%CI (0.70–0.90)	LSS: ICC = 0.85, 95%CI (0.69–0.93)	LSS: ICC = 0.86, 95%CI (0.70–0.94)
		BSS: α = 0.72	BSS: ICC = 0.93, 95%CI (0.88–0.96)	BSS: ICC = 0.93, 95%CI (0.84–0.97)	BSS: ICC = 0.87, 95%CI (0.72–0.94)
			Overall: ICC = 0.89, 95%CI (0.81–0.93)	Overall: ICC = 0.95, 95%CI (0.88–0.98)	Overall: ICC = 0.92, 95%CI (0.82–0.91)
Kim, 2012 [42]	TGMD-2	NR	LSS: ICC = 0.80	NR	NR
			BSS: ICC = 0.75		
			Overall: ICC = 0.78		
Kim S, 2014 [43]	TGMD-2	LSS: α = 0.82	Rater A *vs* Rater B:	NR	LSS: r = 0.90
		BSS: α = 0.73	LSS: ICC = 0.94		BSS: r = 0.85
			BSS: ICC = 0.85		
		Overall: α = 0.87	Overall: ICC = 0.97		
			Rater A *vs* Rater C:		
			LSS: ICC = 0.90		
			BSS: ICC = 0.80		
			Overall: ICC = 0.93		
			Rater B *vs* Rater C:		
			LSS: ICC = 0.91		
			BSS: ICC = 0.77		
			Overall: ICC = 0.92		
			Rater A *vs* Rater B *vs* Rater C		
			LSS: ICC = 0.94		
			BSS: r = 0.92		
			Overall: ICC = 0.96		
Kim C-I, 2014 [44]	TGMD-2	Pre-weighting:	NR	NR	NR
		LSS: α = 0.53			
		BSS: α = 0.68			
		Overall: α = 0.72			
		Post-weighting			
		LSS: α = 0.66			
		BSS: α = 0.53			
		Overall: α = 0.70			
Lopes, 2018 [45]	TMGD-2	LSS: α = 0.46	*Kappa statistic*	NR	*The Bland-Altman method*
		BSS: α = 0.64	The difference between test and retest measure varied between k = 0.7 (moderate consistency) & k = 1 (perfect consistency).		LSS:
		Overall: α = 0.69			95% of agreement ranged 0.85–1.17
					Agreement ratio: 1 (0.08)
					BSS:
					95% of agreement ranged 0.63–1.16
					Agreement ratio: 0.80 (0.13)
					Overall:
					95% of agreement ranged 0.80–1.13
					Agreement ratio: 0.96 (0.09)
Maeng, 2017 [46]	TGMD-3	NR	LSS: ICC = 0.92, 95%CI (0.82–0.98)	Rater A:	NR
			BSS: ICC = 0.93, 95%CI (0.84–0.98)	LSS: ICC = 0.99, 95%CI (0.98–0.99)	
			Overall: ICC = 0.96, 95%CI (0.91–0.99)	BSS: ICC = 0.97, 95%CI (0.90–0.99)	
				Overall: ICC = 0.99, 95%CI (0.95–0.99)	
				Rater B:	
				LSS: ICC = 0.97, 95%CI (0.90–0.99)	
				BSS: ICC = 0.98, 95%CI (0.91–0.99)	
				Overall: ICC = 0.98, 95%CI (0.91–0.99)	
				Rater C:	
				LSS: ICC = 0.99, 95%CI (0.95–0.99)	
				BSS: ICC = 0.97, 95%CI (0.87–0.99)	
				Overall: ICC = 0.98, 95%CI (0.93–0.99)	
				Rater D:	
				LSS: ICC = 0.94, 95%CI (0.76–0.98)	
				BSS: ICC = 0.93, 95%CI (0.73–0.98)	
				Overall: ICC = 0.95, 95%CI (0.81–0.99)	
				Rater E:	
				LSS: ICC = 0.99, 95%CI (0.98–0.99)	
				BSS: ICC = 0.99, 95%CI (0.98–0.99)	
				Overall: ICC = 0.99, 95%CI (0.98–0.99)	
				All raters:	
				LSS: ICC = 0.98, 95%CI (0.96–0.99)	
				BSS: ICC = 0.96, 95%CI (0.94–0.98)	
				Overall: ICC = 0.98, 95%CI (0.96–0.99)	
Mohammadi, 2019 [47]	TGMD-3	LSS: α = 0.85	LSS: ICC = 0.97, 95%CI (0.96–0.98)	LSS: ICC = 0.98, 95%CI (0.98–0.99)	LSS: r = 0.92
		BSS: α = 0.85	BSS: ICC = 0.98, 95%CI (0.97–0.98)	BSS: ICC = 0.99, 95%CI (0.993–0.996)	BSS: r = 0.94
		Overall: α = 0.91	Overall: ICC = 0.98, 95%CI (0.97–0.98)	Overall: ICC = 0.99, 95%CI (0.994–0.997)	Overall: r = 0.95
Palmer, 2016 [33]	TGMD-2	NR	Novice vs expert coders	NR	NR
			LSS: k = -0.001		
			BSS: k = -0.004		
Rintala, 2017 [48]	TGMD-3	NR	LSS: ICC = 0.56	Rater A:	NR
			BSS: ICC = 0.64	LSS: ICC = 0.69	
			Overall: ICC = 0.62	BSS: ICC = 0.77	
				Overall: ICC = 0.75	
				Rater B:	
				LSS: ICC = 0.73	
				BSS: ICC = 0.73	
				Overall: ICC = 0.73	
Simons, 2008 [21]	TGMD-2	LSS: α = 0.82	LSS: r = 1.00	NR	LSS: r_s_ = 0.90
		BSS: α = 0.86	BSS: r = 1.00		BSS: r_s_ = 0.92
		GMQ: α = 0.90	GMQ: r = 1.00		GMQ: r_s_ = 0.98
Valentini, 2008 [32]	TGMD-2	NR	NR	NR	LSS: r = 0.82
					BSS: r = 0.88
Valentini, 2012 [49]	TGMD-2	NR	LSS: ICC = 0.88	ICC = 0.96	LSS: r = 0.83
			BSS: ICC = 0.89		BSS: r = 0.91
					Overall: r = 0.90
Valentini, 2017 [50]	TGMD-3	*Skills-to-test and subtests correlations*:	Rater 1 x Rater 2	LSS: ICC = 0.90	LSS: r = 0.93
		LSS: α = 0.63	LSS: ICC = 0.95	BSS: ICC = 0.85	BSS: r = 0.81
		BSS: α = 0.76	BSS: ICC = 0.97	Overall: ICC = 0.90	Overall: r = 0.90
		Overall: α = 0.74	Overall: ICC = 0.98		
		*Performance-criteria-to-test and subtests correlations*			
		LSS: α = 0.90			
		BSS: α = 0.88			
		Overall: α = 0.93			
Wagner, 2017 [34]	TGMD-3	LSS: α = 0.76	LSS: ICC = 0.88, 95%CI (0.76–0.95)	LSS: ICC = 0.97, 95%CI (0.94–0.99)	LSS: ICC = 0.94, 95%CI (0.91–0.96)
		BSS: α = 0.89	BSS: ICC = 0.97, 95%CI (0.94–0.99)	BSS: ICC = 0.99, 95%CI (0.98–1.00)	BSS: ICC = 0.98, 95%CI (0.97–0.99)
Webster, 2017 [51]	TGMD-3	LSS: α = 0.92	NR	NR	LSS: ICC = 0.97
		BSS: α = 0.95			BSS: ICC = 0.95
		Overall: α = 0.96			Overall: ICC = 0.97

*TGMD*: Test of gross motor development; *LSS*; Locomotor Skills Score; *BSS*; Ball Skills Score; *GMQ*: gross motor quotient; *ASD*: autism spectrum disorder; α: Cronbach’s coefficient alpha; *SEM*: standard error of measurement; Overall: overall gross motor performance; *ICC*: Intra-class Correlation Coefficient; *CI*: Confidence interval; r: Pearson correlation coefficient; *NR*: Not reported; *CVI*: Content validity index; α*: Alpha no specified; k: kappa statistic; r_s_: Spearman correlation.

### Internal consistency

Internal consistency was evaluated in 14 studies (8 TGMD-2 *vs* 6 TGMD-3) (Table 3). Alpha coefficients were calculated in different groups or participants for locomotor skills score (n = 18), ball skills score (n = 18), overall score (n = 12) and GMQ (n = 3). Ten studies reported alpha coefficients between 0.7 and 0.9 for locomotor skills score (4 over 0.9 [17, 36, 50, 51]), 11 for ball skills score (3 over 0.9 [36, 50, 51]), 4 for overall score (3 over 0.9 [17, 50, 51]) and 3 for GMQ (2 over 0.9 [21, 36]).

### Inter-rater reliability

**Fig 2:**
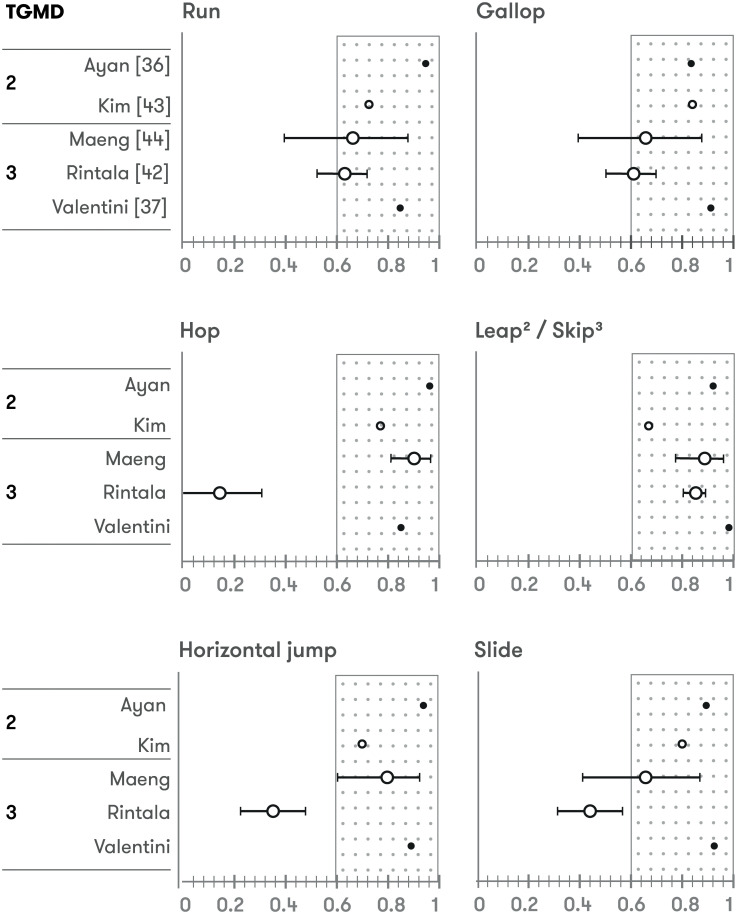
Inter-rater reliability of locomotor skills. *Open circles*: *very good* quality assessment according COSMIN checklist; *Closed circles*: *adequate* quality assessment according COSMIN checklist; *Larger circles*: ICC & 95%CI; *Smaller circles*: ICC.

**Fig 3:**
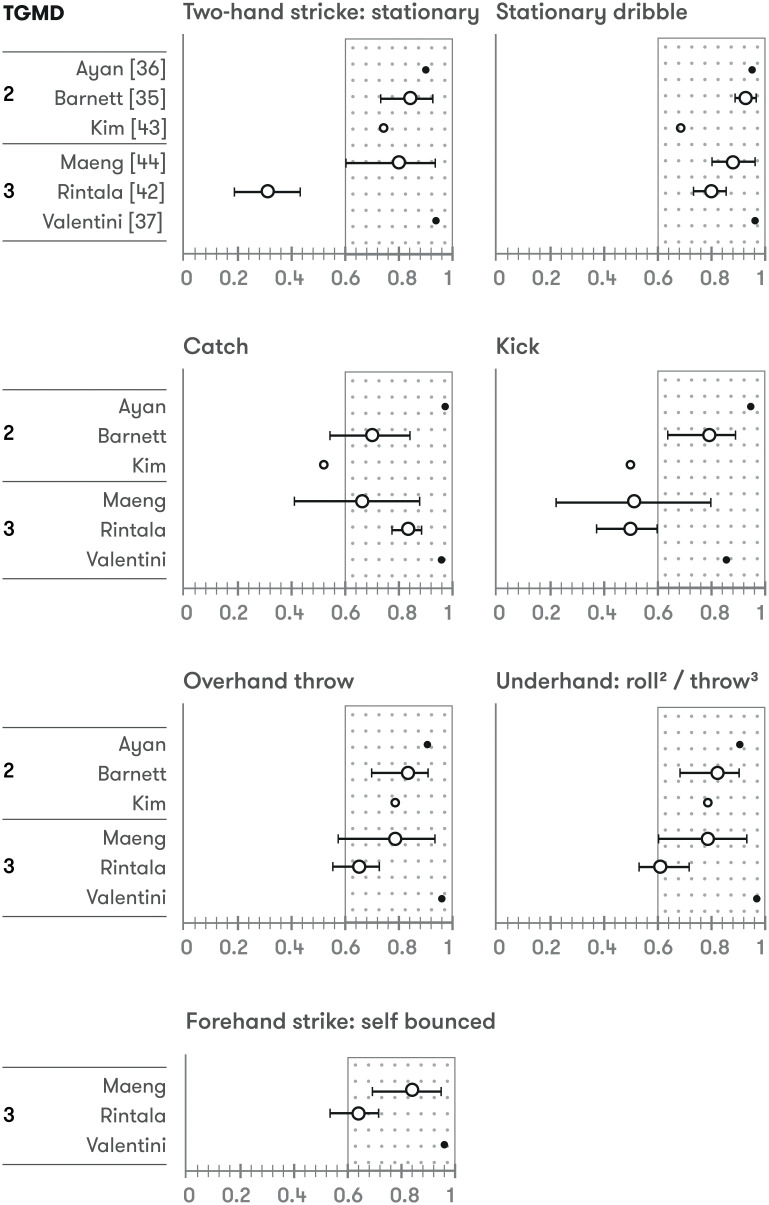
Inter-rater reliability of ball skills. *Open circles*: *very good* quality assessment according COSMIN checklist; *Closed circles*: *adequate* quality assessment according COSMIN checklist; *Larger circles*: ICC & 95%CI; *Smaller circles*: ICC.

Inter-rater reliability was evaluated in 19 studies (12 TGMD-2 *vs* 7 TGMD-3). Three raters were used in 5 studies and 5 raters in 1 study. The rest of them (13) used 2 evaluators. Intraclass correlation (ICC) was calculated in most studies except in four in which Pearson correlation [21], Kappa statistic [33, 45] or content validity index (CVI) [38] were used. ≈70% of the inter-rater statistics reported regarding to locomotor and ball skills’ score, overall score or GMQ were over 0.9. Only two studies shown inter-rater reliability values lower than 0.75 (ICC) [48] or 0.7 (kappa) [33], the last one comparing expert with novice coders. Scores of each individual skill were reported in 6 studies with more than ≈90% of the inter-rater reliability values over 0.6, ≈30% over 0.9 (ICC calculated in all comparisons) [35, 36, 42, 46, 48, 50] (Figs 2 and 3).

### Intra-rater reliability

**Fig 4:**
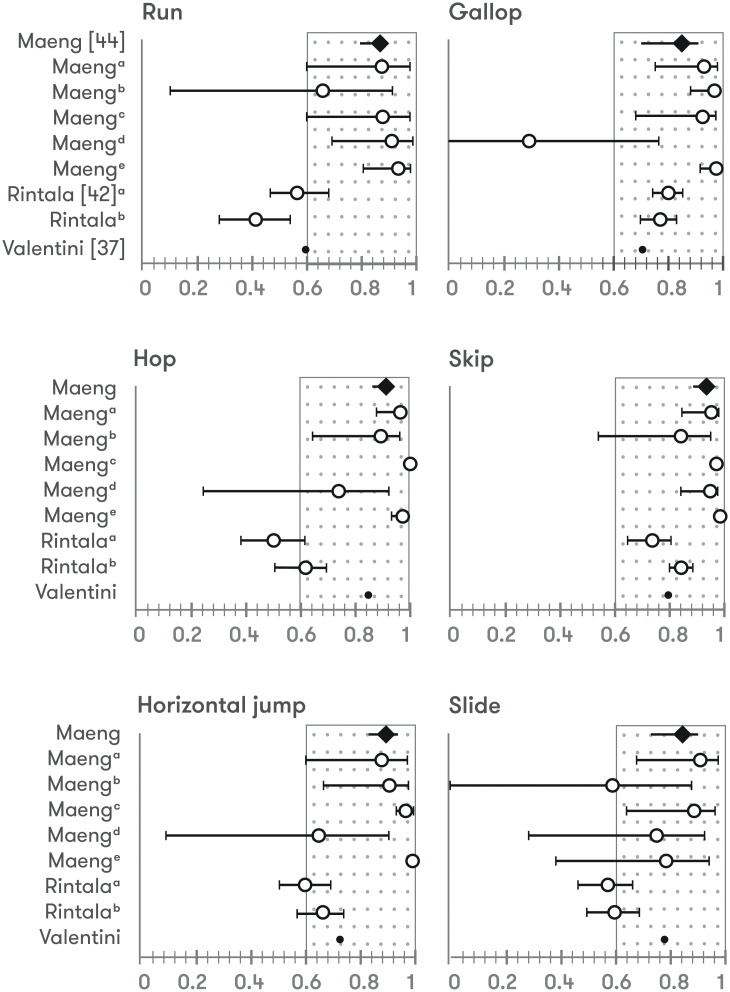
Intra-rater reliability of locomotor skills. ^*a-e*^: Intra-rater reliability of each rater; *Diamonds*: Intra-rater reliability of all raters; *Open circles*: *very good* quality assessment according COSMIN checklist; *Closed circles*: *adequate* quality assessment according COSMIN checklist; *Larger circles*: ICC & 95%CI; *Smaller circles*: ICC.

**Fig 5:**
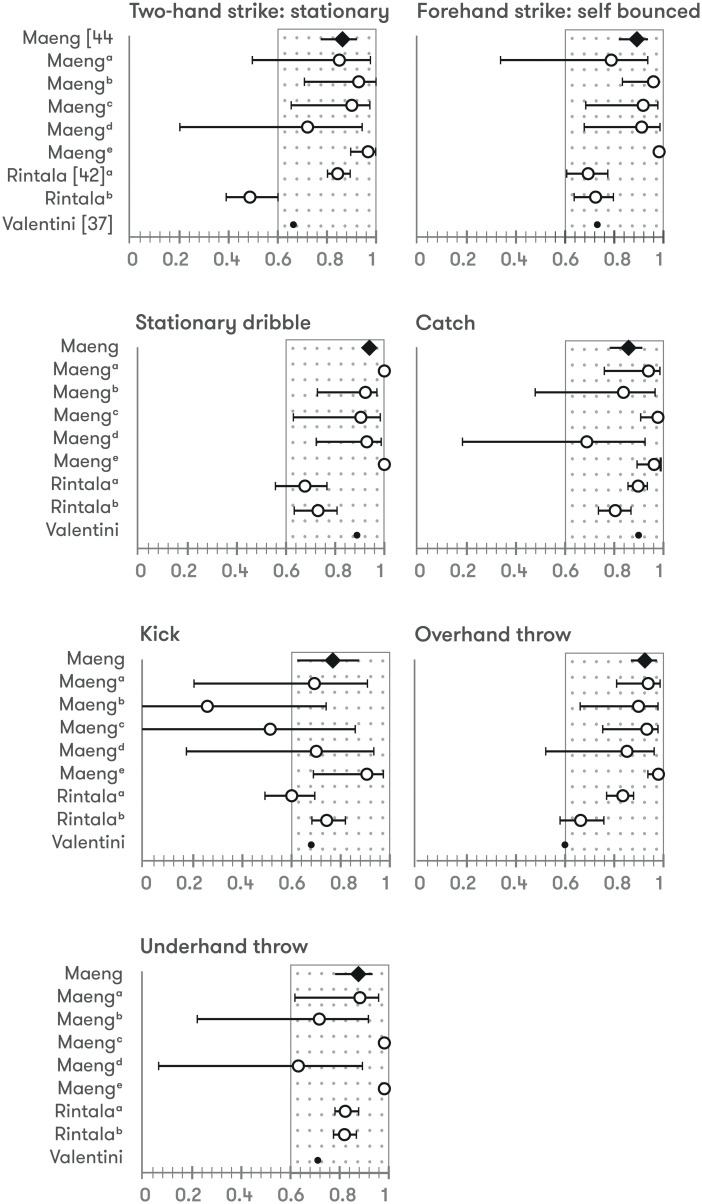
Intra-rater reliability of ball skills. ^*a-e*^: Intra-rater reliability of each rater; *Diamonds*: Intra-rater reliability of all raters; *Open circles*: *very good* quality assessment according COSMIN checklist; *Closed circles*: *adequate* quality assessment according COSMIN checklist; *Larger circles*: ICC & 95%CI; *Smaller circles*: ICC.

Intra-rater reliability was evaluated in 13 studies (6 TGMD-2 *vs* 7 TGMD-3). Two raters were used in 2 studies [39, 48] and 3 [49] and 5 [46] raters in 1 study. One study did not report the number of raters [40], and the remaining studies (8) used 2 evaluators. ICC and CVI were used in 12 and 1 study respectively. ≈85% of the intra-rater statistics shown regarding to locomotor and ball skills’ score, overall score or GMQ were over 0.9, >95% over 0.75. Three studies reported specific data of each locomotor and ball skills, with ≈90% of intra-rater reliability values over 0.6, ≈35% over 0.9 (ICC calculated in all comparisons) [46, 48, 50] (Figs 4 and 5).

### Test-retest reliability

**Fig 6:**
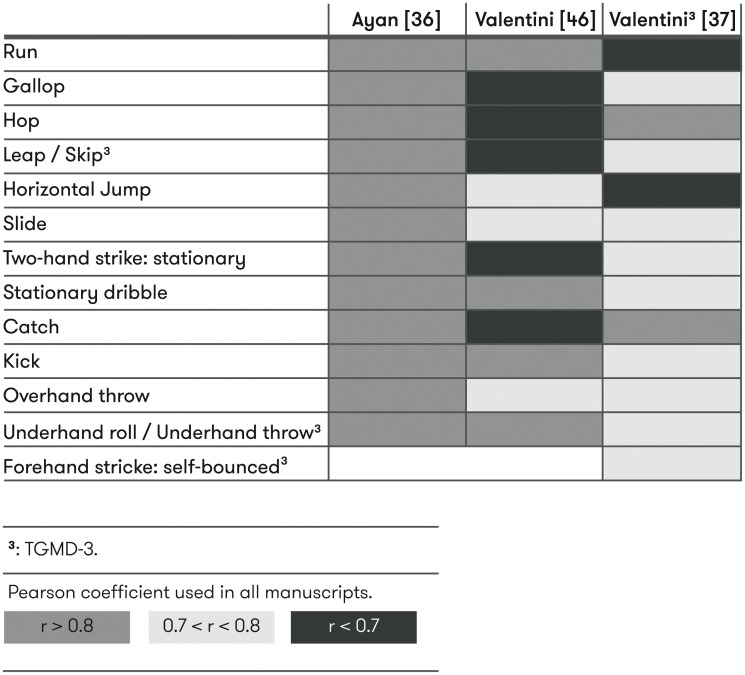
Test-retest reliability of locomotor and ball skills.

Test-retest reliability was evaluated in 15 studies (10 TGMD-2 *vs* 5 TGMD-3). Test-retest reliability was evaluated using ICC (n = 6), Pearson correlation (n = 7), CVI (n = 1) and agreement ratio (n = 1). Reliability of TGMD measured over time showed values over 0.8 in 100% of the evaluations calculated with Pearson correlation, CVI and agreement ratio regarding to locomotor and ball skills’ score, overall score or GMQ. In terms of ICC, more than 95% of values were over 0.75, 40% over 0.9. In three studies test-retest reliability was calculated for each skill with reliability values over 0.8 in ≈50% (Pearson correlation calculated in all comparisons) [36, 49, 50] (Fig 6).

### Children with disabilities

Five studies analysed some reliability measure in children with disabilities: autism syndrome disorder (ASD) (TGMD-3) [17], intellectual disability (TGMD-2) [21, 39, 42] and visual impairment (TGMD-2) [19]. In addition, in the case of ASD children, both protocols, traditional and with visual support were evaluated. Internal consistency was measured in four of the articles with values over 0.7 in all measurements regarding to locomotor and ball scores skills, overall score and GMQ. Inter-rater reliability was evaluated in the five manuscripts, while intra-rater and test-retest reliability were tested in three articles. High reliability was observed with scores over 0.9 in ≈90% of the cases in terms of inter- and intra-rater; ≈70% in test-retest.

### Quality of studies

One study was classified as being of *very good* quality in terms of internal consistency and another one *insufficient*. The rest of the articles were classified as *doubtful*. The item of the COSMIN checklist with lower scores was the one referred to the calculation of statistics for each unidimensional scale or subscale separately. Inter-rater reliability was considered *very good* in 8 studies and *adequate* in other 8 (out of 19 studies). Similar results were found with regard to intra-rater reliability, with 5 studies with *very good* evaluation and 5 with *adequate* (out of 13 studies). Test-retest reliability was classified as *adequate* in 7 studies (out of 15 studies). More detailed results of the COSMIN quality evaluation is shown in Table 4.

**Table 4:** Quality assessment of the studies using the COSMIN checklist.

First author, year	Internal Consistency		Inter-rater	Intra-rater		Test-retest			
	Scales/ subscales	Statistics	Statistics	Time interval	Statistics	Patients stable	Time interval	Test conditions	Statistics
Allen, 2017 [17]	d	v	v	v	v	a	v	v	v
Ayán, 2019 [36]	v	v	a	--		a	v	v	d
Aye, 2017 [37]	--		v	v	v	a	v	a	v
Barnett, 2014 [35]	--		v	--		--			
Cano-Cappellacci, 2015 [38]	--		i	v	i	a	v	a	i
Capio, 2016 [39]	d	v	a	d	a	--			
Estevan, 2017 [40]	d	v	a	d	a	--			
Farrokhi, 2014 [41]	d	i	--	v	a	a	v	a	a
Houwen, 2010 [19]	d	v	v	v	v	a	v	v	v
Kim, 2012 [42]	--		v	--		--			
Kim S, 2014 [43]	d	v	a	--		a	v	a	d
Kim C-I, 2014 [44]	d	v	--	--		--			
Lopes, 2018 [45]	d	v	v	--		a	v	a	i
Maeng, 2017 [46]	--		v	v	v	--			
Mohammadi, 2019 [47]	d	v	a	v	a	a	v	a	d
Palmer, 2016 [33]	--		i	--		--			
Rintala, 2017 [48]	--		v	v	v	--			
Simons, 2008 [21]	d	v	d	--		a	v	a	d
Valentini, 2008 [32]	--		--	--		a	v	a	d
Valentini, 2012 [49]	--		a	d	a	v	v	a	a
Valentini, 2017 [50]	d	v	a	v	a	a	v	a	d
Wagner, 2016 [34]	d	v	a	v	a	a	v	a	a
Webster, 2017 [51]	d	v	--	--		a	v	a	a

*v*: Very good; *a*: Adequate; *d*: Doubtful; *i*: Insufficient.

## Discussion

The TGMD is one of several process-oriented test batteries that purport to assess motor proficiency using visual observation in preschool and primary school-aged children [8, 52]. The purpose of this systematic review was to examine the literature related to the reliability of the TGMD and critically appraise and summarise their results. Generally, this review revealed strong psychometric properties for both TGMD-2 and TGMD-3, suggesting that TGMD variants could be a good choice when opting for a robust test in motor competence testing using product-oriented approaches.

### Internal consistency

Internal consistency refers to the degree to which test components (i.e. skills in TGMD variants) measure the same construct adequately (i.e. subscales and overall score in TGMD variants) [53]. The results from the 14 studies that evaluated internal consistency reliability confirmed, in most cases, good-to-excellent consistency for the TGMD-2 and TGMD-3 total score and GMQ, and acceptable-to-excellent levels of internal consistency in both subscales (locomotor and object control/ball skills), indicating that the instrument seems to be consistent in evaluating the structures related to the subtests and total score in boys and girls [54]. In addition, skills and performance criteria seems to encompass a representation of the same construction [54].

### Inter-rater reliability

Inter-rater reliability shows the agreement or consistency in scores from two or more raters, and is an essential psychometric property when assessing human movement skill proficiency [35]. The results from the 19 studies that evaluated inter-rated reliability confirmed, in most cases, adequate reliability levels and good-to-excellent ICC values for the TGMD-2 and TGMD-3 between raters in locomotor skills score, ball skills score, overall score, and GMQ, with ≈70% of the inter-rater statistics reported over 0.9 and 100% of coefficient values analysed above the defining thresholds of acceptable reliability for observing human movement screening. Only one study showed moderate levels of inter-rater reliability for locomotor and ball skills’ score and overall score (in TGMD-3) [48]; primary due to the large variability observed among three individual skills (hop, horizontal jump, and two-hand strike). The inter-rater reliability values observed in this systematic review were similar to those reported in other product- and process-oriented instruments like Movement Assessment Battery for Children-2nd edition (MABC-2) [54], Bruininks-Oseretsky Test of Motor Proficiency–2nd Edition (BOT-2) [55], Basic Motor Competencies (MOBAK) [56], or Dragon Challenge [57].

### Intra-rater reliability

Intra-rater reliability shows the degree of agreement among repeated evaluations of a test performed by the same rater. This review found excellent ICC values of intra-rater agreement for the TGMD variants in overall score and GMQ, and good-to-excellent in locomotor skills score and ball skills score. In addition, all but one [48] of the included studies reported adequate intra-rater reliability levels above the defining thresholds of acceptable reliability for this systematic review [25, 28]. Similar to inter-rater reliability analysis, only one study showed moderate levels of intra-rater reliability for locomotor skills score, ball skills score and overall score of TGMD-3, primarily due to the large variability observed among five individual skills (run, two-hand strike, slide, hop, and horizontal jump) [48]. Generally, the intra-rater reliability results of the studies included were somewhat higher than those observed for inter-rater reliability, supporting the evidence that is more likely that an evaluator will agree more consistently with him or herself than with other raters [19], which relates to the rater’s subjectivity and discretion [38]. In order to minimise the probability that a rater would remember how he or she scored a specific child’s performance from the previous scoring, the interval between evaluations is considered essential. Indeed, the time gap used in the studies included in this review to reduce memory-influenced bias varies from 12 days [41] to 3 months [48]. In addition, in three of these studies, the interval has not been specified [39, 40, 49]. Consequently, criteria relating to the time interval between tests used in the intra-rater reliability studies analysed seems to be due to an arbitrary chosen. Thus, further research should compare the intra-rater reliability of different TGMD variants using different time intervals to determine the optimal time gap to minimise memory bias.

### Test-retest reliability

Test-retest reliability shows the temporal stability in scores measured by the same rater. Both TGMD variants revealed adequate levels of test-retests reliability, with 100% of the statistics reported above the defining thresholds of acceptable reliability for this systematic review [25, 28]. Specifically, TGMD-2 showed good-to-excellent ICC values of test-retest reliability (assessed in 10 studies) in overall score, GMQ, and ball skills score, and moderate-to-excellent ICC values of test-retest reliability in locomotor skills score. TGMD-3 (assessed in 5 studies) showed excellent ICC values of test-retest reliability in GMQ, and good-to-excellent ICC values in locomotor skills and ball skills. Test-retest reliability values observed in TGMD-2 and TGMD-3 were similar to those reported in other process-oriented instruments that assess individual skills in isolation, such as Victorian FMS Assessment [58].

Familiarisation of the evaluated participants with the testing procedures is an important factor that may influence reliability in a performance test [59]. In this regard, it is important to note that TGMD-2 and TGMD-3 examiner’s manuals indicate that each participant should complete only one familiarisation trial for each skill after verbal description and demonstration of the evaluator [14, 15]. Thus, based on these results, test-retest reliability seems to be consistent regardless of the TGMD variant used and short familiarisation period.

### Cultural and language adaptations

The different TGMD variants are widely used in several countries around the world. However, TGMD was developed for typically developing North American children. Due to the socio-cultural relevance of the subtests and the performance-criteria, several cross-cultural studies have investigated the psychometric properties of TGMD-2 and TGMD-3 in different languages, such as Spanish [36, 38, 40], Persian [47], German [34], or Portuguese [45, 49, 50] and/or cultures [43, 44]. Research conducted in this regard has described high and similar reliability characteristics to the original version, which evidences the clarity of TGMD instructions and the unambiguity of scoring [47].

### Video-vs-live assessment

Although the TGMD examiner’s manual does not assume videotaping assessment [48], most studies included in this review used video-recording evaluations (n = 19). TGMD videotaping evaluation seems to have several advantages as it allows more detailed scrutiny, assists observation of difficult performance criteria with slow-motion replay, and makes it possible to play each performance as many times as needed [48]. In addition, it is less time-consuming in educational settings as test scoring can be done outside classroom time. However, TGMD videotaping evaluation is not always possible due to different ethical considerations or the equipment required [35]. In this respect, it is important to note that the 3 studies that analysed TGMD reliability using live observation showed excellent values of inter-rater [21, 35], test-retest [21, 51], and good-to-excellent internal consistency [21, 51]. Intra-rater reliability was not assessed using live observation in any of the manuscripts included in this systematic review. Thus, TGMD variant reliability seems to be consistent regardless of the type of assessment. However, further research is needed to confirm these findings, comparing the reliability of different TGMD variants using live-versus-video assessment, and the association between rater training and the capacity to carry out live evaluation.

### Rater training

According to the TGMD examiner’s manual, supervised practice is recommended in administering and interpreting motor development tests, with at least three previous assessments before using TGMD in a real situation [14, 15]. However, rater familiarisation, training, and experience in TGMD administration were not systematically reported or described in the studies included in this systematic review. In addition, the academic background of the raters is heterogeneous, varying from graduate students (physical education [35, 36, 48] and sport sciences [36]), master’s students [43, 44], doctoral students [33, 43, 50], physical therapists and physiatrists [37], or paediatric physiotherapists [39]. Previous evidence underscores the need to provide standardised training protocols for coding using process-oriented approaches like TGMD-2 and TGMD-3 for valid and reliable results [46]. However, to the best of our knowledge, only one study analysed scoring differences using TGMD-2 between expert and novice coders [33]. The results showed that novice (undergraduate students in physical education with a two-hour training session on coding process) and expert (doctoral student in motor behaviour with more than 3 years of experience coding the TGMD-2) raters produced significantly different scores except for the kick and the gallop [33], suggesting a need for more extensive training until agreement is obtained. Thus, future research is necessary to explore the effects of providing standardised training protocols for coding TGMD-2 and TGMD-3 data and to determine the minimum training necessary to ensure acceptable reliability levels. In addition, future research should examine the subtest and the performance criteria in which the raters are mostly inconsistent, to paid special attention during familiarisation assessors.

### Children with disabilities

While most of the studies included in this review have analysed reliability in typically developing children, five studies were conducted among children with intellectual disability [21, 39, 42], children with visual impairments [19], and children with ASD [17]. Generally, inter-rater (good-to-excellent), intra-rater (moderate-to-excellent) and test-retest (good-to-excellent), reliability values observed were similar to those reported in typically developing children. Based on these findings, TGMD variants could be considered an appropriate tool to examine FMS in these populations. However, the lower number of studies conducted in children with disabilities opens up an opportunity for future high-quality studies in these and other special populations.

### Reliability of each skill

The reliability of each skill of TGMD-2 and TGMD-3 was evaluated in 7 studies [35, 36, 42, 50, 48, 46, 49]. In general, acceptable levels (ICC ≥ 0.6) of inter-rater reliability were observed for four locomotor skills (run, gallop, leap, and skip) and four ball skills (stationary dribble, overhand throw, underhand roll/throw, and forehand strike), showing moderate-to-excellent ICC values. However, the remaining three locomotor (hop, horizontal jump, and slide) and three ball skills (two-hand strike, catch, and kick) showed conflicting levels of inter-rater reliability. Differences in reliability between skills could be a reflection of the difficulty involved in assessing some skill components or performance criteria and the need to improve clarity in their scoring and interpretation.

Intra-rater reliability of individual skills were somewhat higher than those observed for inter-rater reliability, with seven skills (skip, horizontal jump, forehand strike, stationary dribble, catch, overhand throw, and underhand throw), showing moderate-to-excellent ICC values. The remaining six skills (run, gallop, hop, slide, two-hand strike, and kick) revealed conflicting levels of intra-rater reliability, which may reflect the need for more intensive training on the performance criteria evaluation for these specific skills [46]. It is important to note that the three studies that analysed intra-rater reliability of each skill used TGMD-3 version. Further research seems to be necessary to analyse this in TGMD-2, which is the most used variant of the test in scientific context.

Three studies evaluated test-retest reliability of each individual skill of TGMD-2 [36, 49] and TGMD-3) [50]. Several discrepancies were found in this regard in studies which used TGMD-2. Ayan et al [36] showed acceptable test-retest reliability levels (Pearson correlation ≥ 0.7) in all skills; however, Valentini [49] in seven skills (run, horizontal jump, slide, stationary dribble, kick, overhead throw and underhand roll). In the case of TGMD-3, only run and horizontal jump were skills with low test-retest reliability values, which might reflect higher levels of temporal stability in TGMD-3 than TGMD-2 [50]. However, due to the low number of studies, to further explore this area, future research may be needed to confirm these findings.

### Methodological quality

Fourteen studies evaluated internal consistency, and only one was classified as being of *very good* quality [36]. Any of the remaining studies did not calculated or expressed statistics for each unidimensional scales or subscales as it is highlighted [25]. That also involves calculation of Cronbach’s alpha for each skill. In terms of inter-rater, intra-rater and test-retest reliability, the statistical methods item was the one which penalized the most. According with the COSMIN checklist, only using ICC (showing formula or model used) for continuous scores or kappa for dichotomous/nominal scores is possible to achieve a *very good* mark in this item [25]. However, previous studies suggested that coefficient of variation might be used in this regard with great applicability [60, 61]. Even so, most of manuscript in which inter- and intra-rater were evaluated were classified as being of *very good*/*adequate* quality.

Pearson correlation was used in the majority of the manuscripts in order to evaluate test-retest reliability. Nevertheless, this statistic is not considered the most suitable to assess reliability [25, 29, 62]. In addition, evidence that patients were stable between both evaluations is mandatory to be classified as *very good*. Since it might consider highly probable that children were stable during the evaluation, but no evidence was often provided, most manuscripts were classified in this item as *adequate*. Due to these rigorous and exigent methodological aspects in terms patients and statistics, no studies were classified as being of *very good* quality.

### Limitations

A first limitation of this systematic review can be identified in the specific eligibility criteria that excluded the so-called grey literature. Thus, relevant publications could have been not included in this synthesis (i.e. monographs, conference abstracts, dissertations and theses). In addition, only publications in English, Spanish, or Portuguese that primary investigated reliability were selected. It can be assumed that significant articles could have been published in other languages. Another limitation was the absence of any form of meta-analysis in this systematic review due to the broad variety of statistical procedures employed to determine reliability and the heterogeneity of participants. Finally, as TGMD-3 variant is a relatively new test, the number of included studies that analysed psychometric properties of this version was significantly lower than TGMD-2.

## Conclusions

A total of 23 studies were considered in this systematic review. Overall, the results of this systematic review indicate that, regardless of the variant of the test and the type of assessment (i.e. live vs. video), the TMGD has moderate-to-excellent internal consistency, good-to-excellent inter-rater reliability, good-to-excellent intra-rater reliability, and moderate-to-excellent test-retest reliability. Furthermore, reliability seems to be high both in typically developing children and children with disabilities; however, the lower number of studies in special populations reveals the need of further high-quality studies. Since there is no gold standard for assessing FMS, TGMD variants could be appropriate when opting for a psychometrical robust test. However, standardized training protocols for coding TGMD variants seem to be necessary both for researchers and practitioners in order to ensure acceptable reliability. Nevertheless, the optimal training protocol requires further study. Finally, due to the few high-quality studies in terms of internal consistency, it would be recommend that further studies in this field refer to the COSMIN checklist to improve their quality.

## Supporting information

S1 File Research syntax. (DOCX)

S1 Checklist (DOC)
