# Supplementary material for: Reliability of the test of gross motor development: A systematic review
Source: PLoS One. 2020 Jul 16;15(7):e0236070. doi: 10.1371/journal.pone.0236070 (PMC7365594; doi:10.1371/journal.pone.0236070)
Supplement: S1 File — (DOCX) [file pone.0236070.s001.docx]

**S1 File**

**The following research syntax was employed:**

**Block 1:** Motor development

“Fundamental motor skills” OR

“Fundamental motor skill proficiency” OR

“Fundamental movement skills” OR

“Fundamental movement skill proficiency” OR

“Motor skills” (MeSH)

*AND*

**Block 2:** TGMD

“Test of gross motor development” OR

tgmd

*AND*

**Block 3:** Tests & results

“Reliability and validity” (MeSH) OR

“Validity and reliability” (MeSH) OR

“Reproducibility of results” (MeSH) OR

“Reproducibility of findings” (MeSH) OR

Reliability OR

Validity OR

Reproducibility OR

Repeatability
